# Radix Codonopsis: a review of anticancer pharmacological activities

**DOI:** 10.3389/fphar.2024.1498707

**Published:** 2025-01-07

**Authors:** Cai-Yue Liu, Zheng Li, Fan-E. Cheng, Yi Nan, Wei-Qiang Li

**Affiliations:** ^1^ Ningxia Medical University, Ningxia of Traditional Chinese Medicine, Yinchuan, China; ^2^ Key Laboratory of Ningxia Minority Medicine Modernization, Ministry of Education, Ningxia Medical University, Yinchuan, China

**Keywords:** Radix Codonopsis, tumor, traditional Chinese medicine, mechanism, pharmacology

## Abstract

Radix Codonopsis (Dangshen), derived from the dried root of plants in the Campanulaceae family, is a widely used Chinese herbal medicine. It is renowned for its pharmacological effects, including tonifying the middle qi, invigorating the spleen, benefiting the lungs, enhancing immunity, and nourishing the blood. Codonopsis extract is frequently incorporated into health products such as tablets and capsules, making it accessible for daily health maintenance. Additionally, it is commonly used in dietary applications like soups, teas, and porridges to nourish qi, enrich blood, and promote overall vitality. In recent years, increasing attention has been given to the anti-cancer potential of Radix Codonopsis. Studies have identified key active components such as luteolin, stigmasterol, polyacetylenes, lobetyolin, and glycitein, which exhibit anti-tumor properties through mechanisms like inhibiting cancer cell growth and proliferation, suppressing epithelial-mesenchymal transition (EMT), and inducing apoptosis. This review highlights the research progress on Radix Codonopsis, including its active constituents, anti-cancer mechanisms, and its role in the convergence of medicine and food in modern life. By doing so, it aims to provide valuable insights and references for future scientific studies and clinical applications of Radix Codonopsis.

## 1 Introduction

According to the latest cancer statistics of the International Agency for Research on Cancer (IARC), nearly 20 million new cancer cases were reported worldwide in 2022, and this number is expected to exceed 35 million by 2050 (Bray et al., 2024). These figures underscore that cancer remains a major global public health issue. Cancer cells have the ability to invade surrounding tissues and organs and can spread to other parts of the body through hematogenous or lymphatic metastasis. Additionally, many cancers do not present obvious symptoms in their early stages, making early detection difficult. As a result, they are often diagnosed at more advanced stages, complicating treatment efforts. Current cancer treatments primarily involve surgery and chemotherapy; however, chemotherapy is frequently associated with significant side effects that can substantially affect patients’ quality of life. Thus, there is a pressing need for more effective treatment options that minimize side effects and enhance the efficacy of chemotherapy.

Herbal medicine is a treasure trove of nature, and thousands of plants are used in medicine (Wang et al., 2016; Zeng et al., 2020; Gao et al., 2022), including many natural herbal extracts. These extracts are widely used in pharmaceutical research and drug development and have a variety of medical values (Jiang et al., 2018; Yi-wen et al., 2018; He et al., 2023). In addition, herbal remedies have great potential for the treatment of many diseases, including oncological diseases, and modern scientific research is gradually revealing their mechanisms of action, providing important support for the modernization and internationalization of traditional Chinese medicine (Li et al., 2022; Ruan et al., 2023). Radix Codonopsis, a commonly used tonic herb in traditional Chinese medicine (TCM), is recognized for its ability to invigorate the spleen and lungs, nourish blood, and generate body fluids. The root of Codonopsis pilosula is the primary medicinal part, and its chemical composition is complex, containing various biologically active substances, such as alkaloids, terpenoids, flavonoids, lignins, steroids, and sugars. These components confer a range of pharmacological effects, including neuroprotective properties, regulation of blood glucose and lipids, immune system modulation, digestive system support, improvement of blood circulation, and antibacterial and antiviral effects (Yang et al., 2024). In terms of anti-cancer potential, Radix Codonopsis has shown significant promise (Chen et al., 2018; Xin et al., 2012). Clinically, Radix Codonopsis is used to treat a variety of tumors, such as lung cancer, gastric cancer, liver cancer, pancreatic cancer, colorectal cancer, and so on (Zhao et al., 2024).

**FIGURE 1:**
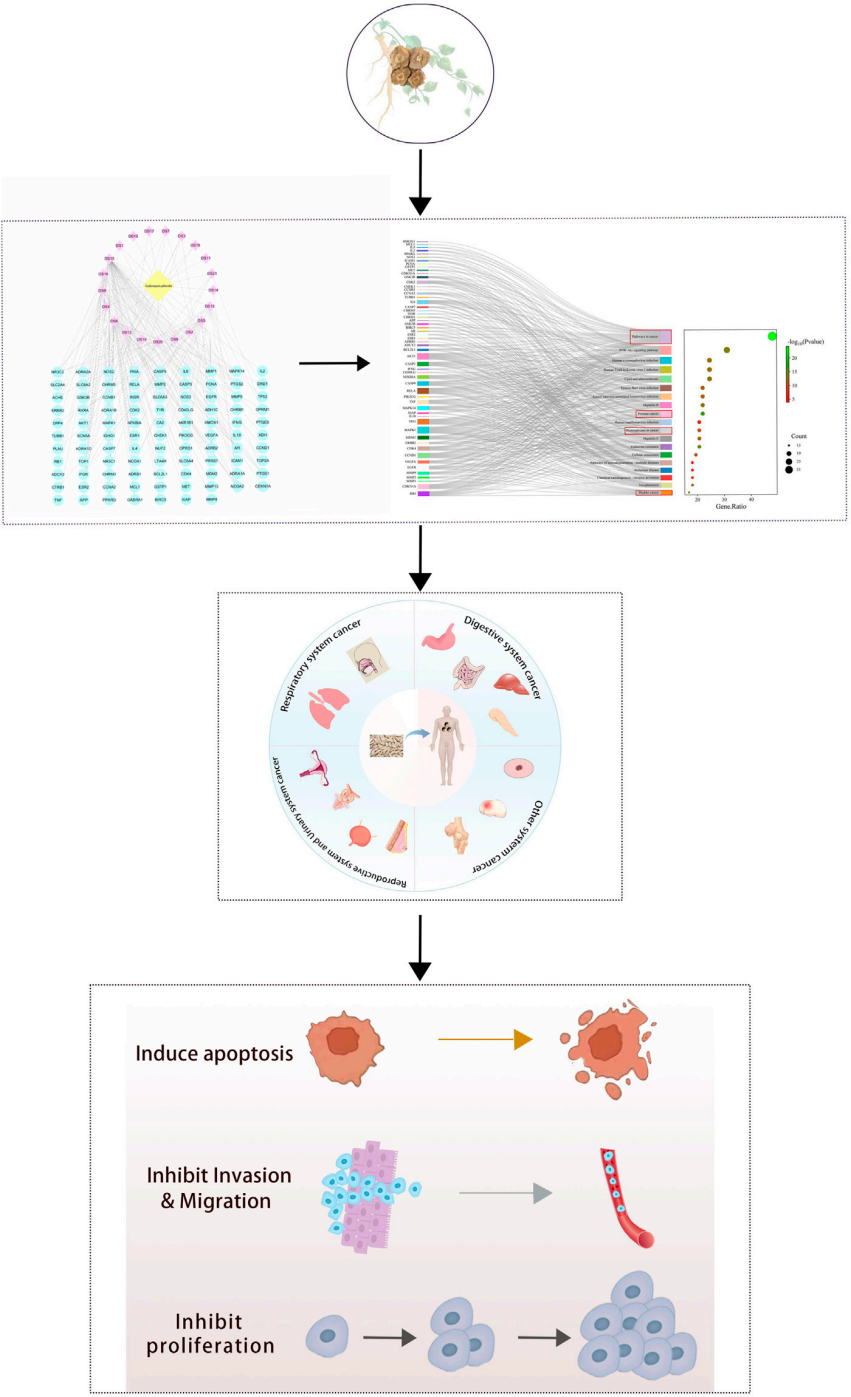
General flow chart of the studies in this review.

This paper provides a comprehensive review of the anticancer effects of Radix Codonopsis, aiming to offer insights for its clinical application in cancer treatment. An overview of the research flow is presented in Figure 1.

## 2 Network diagram of anti-tumor effect of Radix Codonopsis

Network Pharmacology is a multidisciplinary technology that integrates bioinformatics, network science, and pharmacology. It is a method for studying drugs and their mechanisms of action, especially in understanding the characteristics of multi-component, multi-target, and multi-pathway of TCM (Wang et al., 2021). By constructing and analyzing biological molecular networks, we can understand and predict the interactions between drugs and organisms (Noor et al., 2022; Cheng et al., 2024). In cancer research, it can identify key regulatory nodes through the analysis of cancer-related biomolecular networks, which may be potential therapeutic targets. This helps to understand the complex signaling pathways of cancer and provides a theoretical basis for the development of novel anti-tumor drugs targeting multiple targets.

### 2.1 Acquisition of ingredients and targets of Radix Codonopsis

The chemical composition information of Codonopsis pilosula was searched in TCMSP database (https://old.tcmsp-e.com/tcmsp.php (accessed on June 5, 2024)) with “Dangshen” as the key word. The active ingredients of Radix Codonopsis were screened according to the criteria of oral bioavailability (OB ≥ 30%) and drug class (DL ≥ 0.18), and the results were exported. Then use the RelateTargets column of TCMSP platform to collect relevant targets. For components that cannot successfully search for targets, the Canonical SMILE sequence of compounds can be queried through PubChem (https://pubchem.ncbi.nlm.nih.gov), and the target can be predicted using SwissTargetPrediction database (https://www.swisstargetprediction.ch). Finally, 21 active ingredients and 97 targets were obtained.

### 2.2 PPI network construction and bioinformatics analysis

**FIGURE 2:**
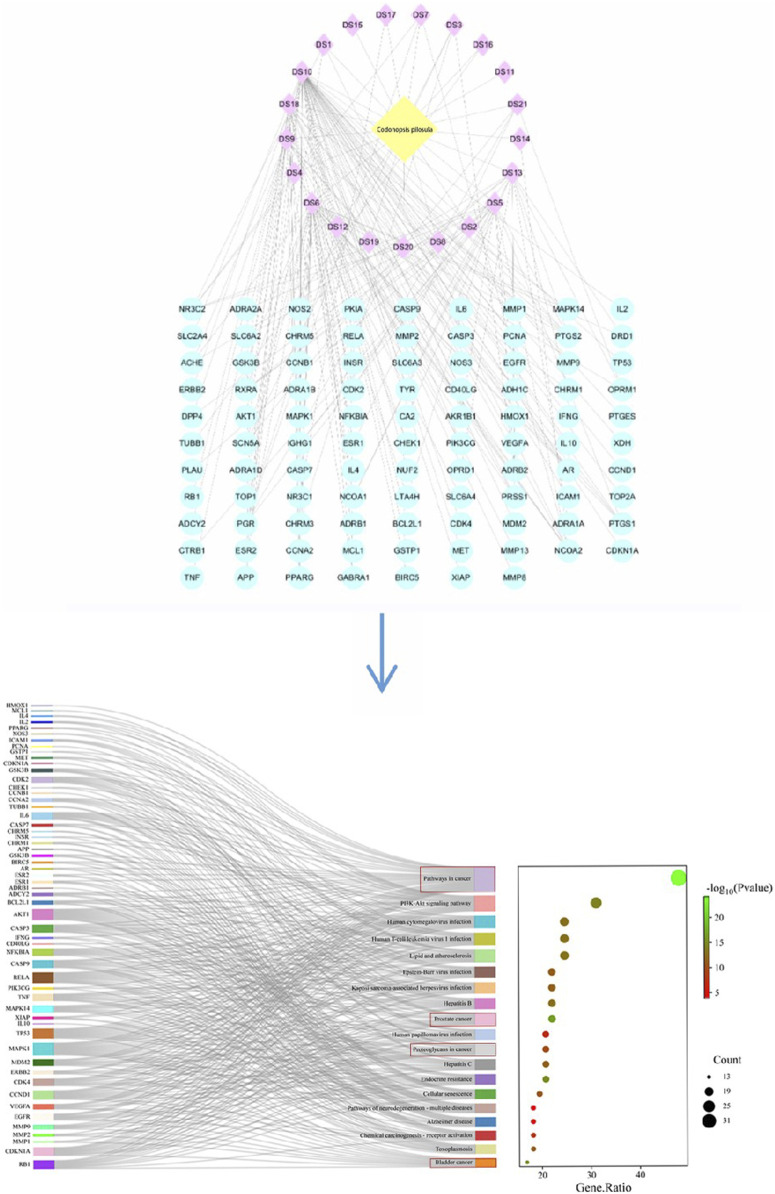
The active ingredient target figure and the sankey diagram of Codonopsis pilosula.

The obtained target genes were imported into the STRING database (https://stringdb.org/(accessed on June 11, 2024)), and the Orgnism was set to “*Homo sapiens*” to obtain the PPI network graph, which was saved in the file format of “tsv” file format. The obtained results were imported into the network visualization tool Cytoscape 3.9.1 to show the network diagrams of the interactions between the 21 active ingredients and 97 corresponding genes. Pathway enrichment analysis of the 97 target genes was then performed using the DAVID database (https://david.ncifcrf.gov (accessed June 12, 2024)): by selecting “GENE SYMBOL” and “HUMAN” and submitting the data, we selected “*Homo sapiens*” and clicked “KEGG Pathways” to download the pathway files. Finally, the enrichment results were plotted on the online Microbiology website (https://www.bioinformatics.com.cn/login/). The results showed that the major active components of Codonopsis had significant pharmacological effects in cancer-related pathways, such as prostate cancer and bladder cancer, as shown in Figure 2. It indicates that Codonopsis pilosula has good application value in anticancer.

## 3 Compound attributes of Radix Codonopsis

Radix Codonopsis, a perennial herb from the Campanulaceae family, contains a diverse range of bioactive compounds, each contributing to its pharmacological effects. These compounds have found wide applications in various therapeutic areas. The basic information on the effective components of Radix Codonopsis is summarized in Table 1.

**TABLE 1:** The basic information of codonopsis pilosula active ingredients.

Active ingredients	Compound category	Mol ID	Class	CAS	OB (%)
poriferasta-7,22E-dien-3beta-ol	Terpenoids	MOL001006	terpenoids	481-18-5	42.98
Perlolyrine	Alkaloids	MOL002140	alkaloids	29700-20-7	65.95
Diop	Alkaloids	MOL002879	alkaloids	25103-50-8 27554-26-3 41375-90-0 71097-28-4 1330-91-2	43.59
ZINC03978781	——	MOL003036	—	19716-26-8	43.83
Stigmasterol	Steroids	MOL000449	steroids	83-48-7	43.83
7-Methoxy-2-methyl isoflavone	Flavonoids	MOL003896	flavonoids	19725-44-1 82517-12-2	42.56
Spinasterol	Steroids	MOL004355	steroids	481-18-5	42.98
Chrysanthemaxanthin	——	MOL004492	—	26989-20-8 27780-11-6	38.72
Frutinone A	——	MOL005321	—	38210-27-4	65.9
luteolin	Flavonoids	MOL000006	flavonoids	491-70-3	36.16
Taraxerol	Terpenoids	MOL006554	terpenoids	22076-46-6 127-22-0	38.4
stigmast-7-enol	Steroids	MOL006774	steroids	481-19-6 6869-99-4 119626-74-3	37.42
3-beta-Hydroxymethyllenetanshiquinone	——	MOL007059	—	—	32.16
methyl icosa-11,14-dienoate	Esters	MOL007514	esters	2463/2/7	39.67
5alpha-Stigmastan-3,6-dione	Steroids	MOL008391	steroids	—	33.12
7-(beta-Xylosyl)cephalomannine_qt	——	MOL008393	—	—	38.33
Daturilin	Steroids	MOL008397	steroids	111950-78-8	50.37
glycitein	Flavonoids	MOL008400	isoflavonoids	40957-83-3	50.48
Spinoside A	Terpenoids	MOL008406	terpenoids	524-40-3	39.97
(8S,9S,10R,13R,14S,17R)-17-[(E,2R,5S)-5-ethyl-6-methylhept-3-en-2-yl]-10,13-dimethyl-1,2,4,7,8,9,11,12,14,15,16,17-dodecahydrocyclopenta [a]phenanthren-3-one	——	MOL008407	—	6869-99-4	45.4
11-Hydroxyrankinidine	Alkaloids	MOL008411	alkaloids	122590-03-8	40

### 3.1 Flavonoids

Flavonoids are a large class of secondary metabolites commonly found in plants, distributed across various plant tissues. These compounds exhibit a wide range of biological and pharmacological activities, including antioxidant (Procházková et al., 2011), anti-inflammation (Serafini et al., 2010), anti-cancer (Dobrzynska et al., 2020), cardiovascular protection (Mladenka et al., 2010), anti-virus and anti-bacteria (Khater et al., 2019), and are used to treat and prevent various diseases. lavonoids are found in foods such as tea, coffee, soybeans, onions, and apples (Serafini et al., 2010). They belong to phenylpropanoid cycloheptene compounds with the basic structure of 2-phenylchromenone. This basic structure is composed of two benzene rings connected by a three-carbon chain to form a C6-C3-C6 skeleton (Preet et al., 2023). Given the structural diversity and complexity of flavonoids, they are a focal point of research in both phytochemistry and medicinal chemistry. Ongoing studies continue to explore their mechanisms of action in health and disease, as well as their future therapeutic potential.

### 3.2 Alkaloids

Alkaloids are nitrogen-containing organic compounds, widely distributed in the plant kingdom, known for their biological activity (Bhambhani et al., 2021). Plants such as the opium poppy, Catharanthus roseus, and Ephedra sinica are important alkaloid sources. Alkaloid research has expanded to encompass their biosynthetic pathways (Kishimoto et al., 2016), pharmacokinetics (Ziegler and Facchini, 2008), chemical structure, biological activity (Bhambhani et al., 2021) and potential applications in various fields. Alkaloids are used in clinical settings as pharmaceutical raw materials, such as morphine and its derivative, codeine, which serve as potent analgesics (Chida, 2011). Quinine, another alkaloid, is a primary treatment for malaria (Shanks, 2016). In addition, alkaloids like camptothecin and vincristine are used in cancer treatment (Patel et al., 2022). Vincristine is usually used to treat lymphoma and leukemia (Li et al., 2007). Camptothecin is commonly used in the treatment of ovarian, colorectal, and lung cancers (Al-Ghazzawi, 2019). Berberine, a notable isoquinoline alkaloid, is widely used for treating gastrointestinal diseases such as bacterial diarrhea and infections, while exhibiting antibacterial, anti-inflammatory, hypoglycemic, and anti-tumor properties (Song et al., 2020).

### 3.3 Steroids

Steroids are organic compounds with a four-ring structure, found in a variety of natural sources, including phytosterols, bile acids, cardiac glycosides, steroidal saponins, and steroidal alkaloids. Steroids are integral to various medical applications due to their unique molecular structure, which contains a parent nucleus known as “cyclopentane polyhydrophenanthrene” (Mensah-Nyagan et al., 2009). This structure confers a wide array of biological activities. In medicine, steroid compounds often mimic or regulate natural hormones in the human body, such as adrenal cortical hormones and sex hormones (Gupta et al., 2013). These compounds are used in hormone replacement therapies and contraceptives. For instance, androgens are used to treat menopausal symptoms, while estrogens and progesterones are key components of oral contraceptives (Frank and Schneider, 2013). Additionally, some steroidal compounds possess anti-tumor effects. For example, steroidal saponins can induce apoptosis, promote autophagy, inhibit tumor cell migration, and cause cell cycle arrest, thereby exhibiting anti-cancer properties (An et al., 2022). Steroids also play roles in anti-inflammatory processes, immune regulation, blood pressure control, and cardiovascular health (Racette et al., 2015; Giles et al., 2018; Li et al., 2021).

### 3.4 Terpenoids

Terpenoids are a vast class of natural organic compounds, either open-chain or cyclic, derived from isoprene units. As the largest group of plant secondary metabolites, they are central to various biological processes and widely used across industries such as medicine, food, and cosmetics (Bouvier et al., 2005; Nagegowda and Gupta, 2020). Terpenoids exhibit a broad spectrum of biological activities, including antibacterial, anti-inflammatory, anti-tumor, anti-malarial effects, coronary artery dilation, and immune system enhancement. Their specific biological effects are intricately linked to their unique chemical structures. For example, artemisinin, a well-known terpenoid lactone, is extensively used as an anti-malarial agent (O’Neill et al., 2010). Andrographolide, a diterpene derived from Andrographis paniculata, possesses potent antibacterial and anti-inflammatory properties (Zhang et al., 2021). Paclitaxel, another diterpenoid, is widely employed in the treatment of breast and ovarian cancers (Zhu and Chen, 2019). Tanshinone, a diterpene found in Salvia miltiorrhiza, provides cardiovascular protection (Li et al., 2020). Ginsenoside, a triterpenoid, is renowned for its immune-boosting effects (Choi and Kim, 2023). In addition, terpenoids exhibit neuroprotective activity, making them valuable in the treatment of neurodegenerative diseases (Manju and Bharadvaja, 2024). Given their extensive range of biological activities, terpenoids have become a critical focus in pharmaceutical research and development. As studies continue to explore their therapeutic potential, we can expect the emergence of novel drugs based on terpenoid compounds in the future.

## 4 Chemical structure formula of main anti-cancer components of Radix Codonopsis

**FIGURE 3:**
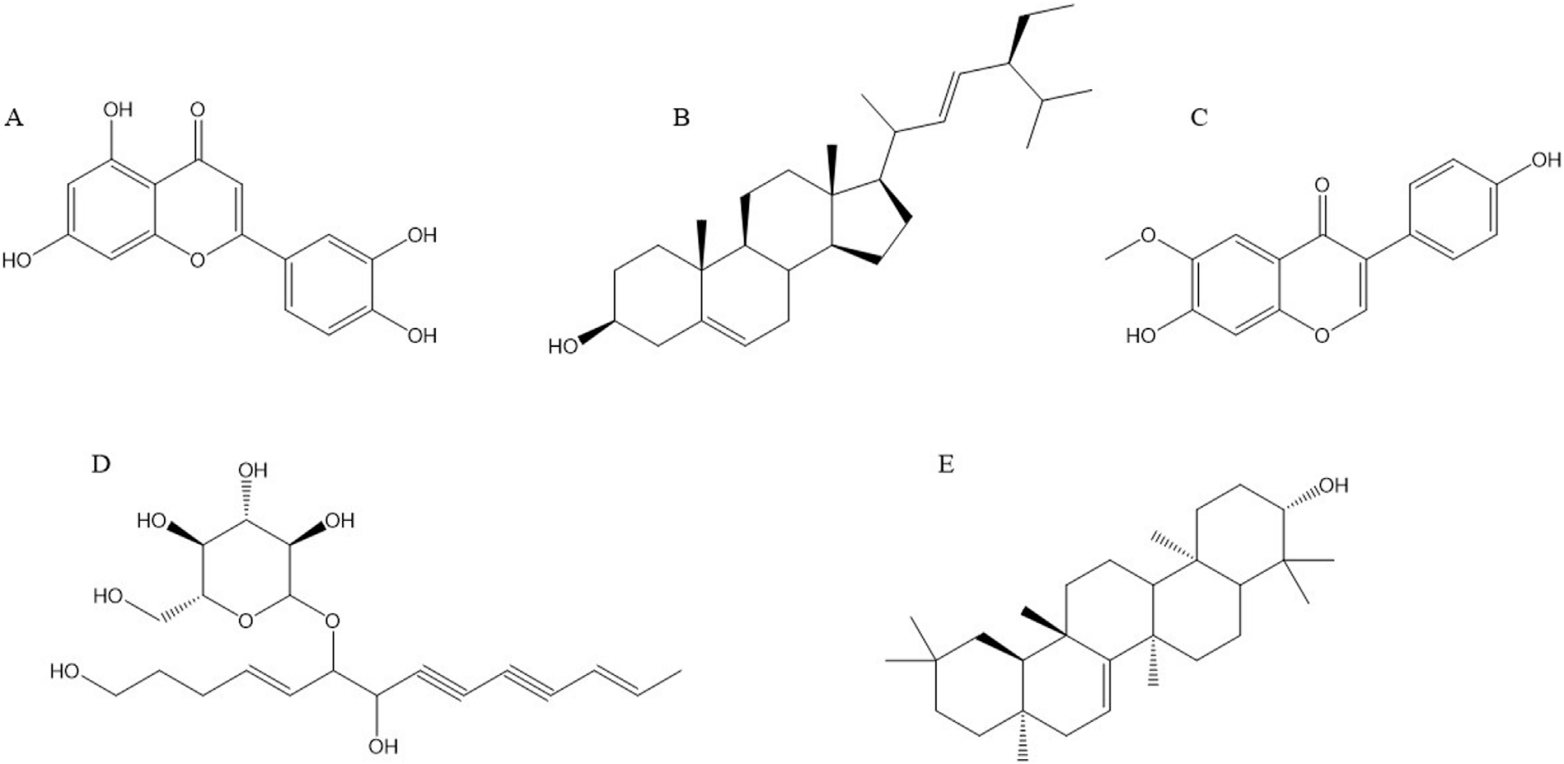
Structural formulae of representative compounds: **(A)** Luteolin, **(B)** Stigmasterol, **(C)** Glycitein, **(D)** Lobetyolin, **(E)** Taraxerol.

Recent studies have demonstrated that Radix Codonopsis contains a variety of active ingredients with significant anticancer properties. The key anticancer components include luteolin, stigmasterol, lobetyolin, glycitein, taraxerol, and others. These compounds exert their anti-cancer effects through various mechanisms, such as inducing tumor cell apoptosis and inhibiting tumor cell proliferation. The chemical structures of these active ingredients are depicted in Figure 3.

## 5 The anti-cancer effect of Radix Codonopsis in various systems

**FIGURE 4:**
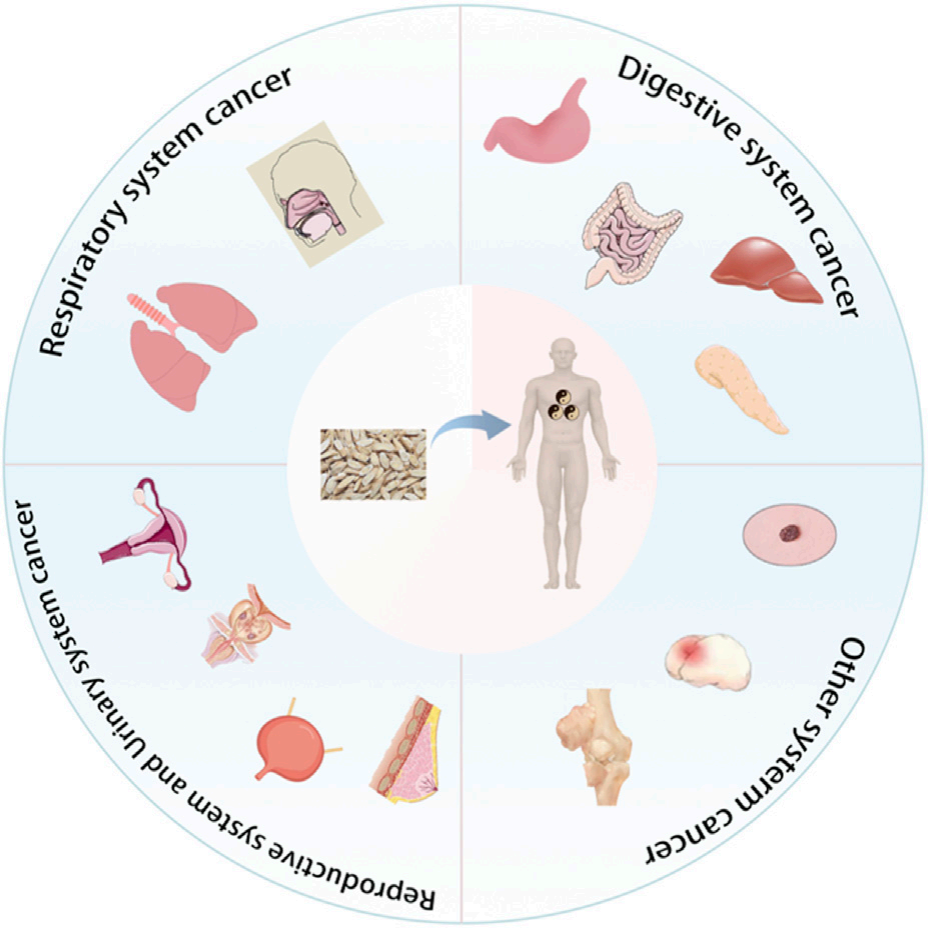
Radix Codonopsis plays a role in cancer of various systems.

The anticancer effects of Radix Codonopsis’s active ingredients involve multiple systems, including the respiratory, digestive, and reproductive systems (see Figure 4). The mechanisms of action include direct induction of tumor cell apoptosis, inhibition of tumor cell proliferation, and anti-metastatic effects, as summarized in Table 2.

**TABLE 2:** Effect and mechanism of Codonopsis pilosula on respiratory system tumors, digestive system tumors, genitourinary system tumors and other system tumors.

System	Cancer	Compound	Relevant mechanism	Passway/Protein	Reference
Respiratory system cancer	lung cancer	luteolin	Inhibit migration and invasion	interfering in the PI3K/Akt-NF-κB-Snail pathway to attenuate TGF-β1-induced epithelial-mesenchymal transition	Chen et al. (2013)
		Polyacetylenes	Induced apoptosis	inactivate of the Ras/PI3K/AKT pathway and ameliorated lung dysbiosis	Wang et al. (2022)
		luteolin	Inhibition of proliferation	Downregulates TAM RTK	Lee et al. (2017)
	non-small cell lung cancer	luteolin	Induced G2/M stagnation and inhibited migration	Decreased the expression of melanoma 2 (AIM2), induced G2/M phase arrest in NSCLC and inhibited EMT	Yu et al. (2019)
		luteolin	Inhibit migration and invasion	inhibiting the expression of integrin β1 and FAK to inhibit hypoxia-induced EMT	Ruan et al. (2012)
		luteolin	Induced apoptosis	activate a p38/ROS/caspase cascade	Cho et al. (2015)
		luteolin	Induced apoptosis	The protein expression level of Sirt1 in NCI-H460 cell line was inhibited	Ma et al. (2015)
		luteolin	Inhibition of proliferation	Caspase activation and extracellular signal-regulated kinase/Akt inhibition	Kim et al. (2007)
	Lewis lung cancer	luteolin	Pro-apoptotic and anti-migration	Activate the MEK/ERK signaling pathway	Meng et al. (2016)
	lung adenocarcinoma	Stigmasterol	Induced apoptosis and protective autophagy	Inhibition of Akt/mTOR pathway	Zhao et al. (2021)
Digestive system cancer	Gastric Cancer	taraxerol	Induced apoptosis	Inhibit the activation of PI3K/AKT signaling pathway	Huo et al. (2022)
		luteolin	Inhibit angiogenesis	Inhibition of Notch1-VEGF signaling pathway	Zang et al. (2017)
		luteolin	Induced apoptosis	Upregulated expression of miR-34a and downregulated expression of Bcl-2	Wu et al. (2015)
		luteolin	Inhibit proliferation, invasion and promote apoptosis	Inhibition of cMet/Akt/ERK signaling pathway	Lu et al. (2015)
		lobetyolin	Inhibit proliferation and promote apoptosis	Downregulation of ASCT2 expression induces ROS accumulation and mediates the AKT/GSK3β/c-Myc pathway	Cheng et al. (2023)
		glycitein	Induced cell apoptosis and G1/G3 cell cycle arrest	Ros-related MAPK/STAT3/NF-κB signaling pathway	Zang et al. (2019)
		luteolin	Induced apoptosis	Activate AMPK	Hwang et al. (2011)
	Liver cancer	Stigmasterol	Induced apoptosis	Remodeling gut microbiota and increase the proportion of IFN-γ+ CD8+ T cells and Treg cells in both the intestinal mucosa and tumor tissues	Huo et al. (2024)
		CPP1a、CPP1c	Inhibit migration and induce apoptosis	Upregulation of Bax/Bcl-2 ratio and activation of caspase-3	Bai et al. (2018)
		Codonopsis pilosula polysaccharide solution (CPP)	Inhibit proliferation, migration and induce apoptosis	inhibit the expression of CDK1/PDK1/β-catenin signaling axis factors	Li et al. (2024)
		luteolin	Inhibit proliferation, migration and induce apoptosis	Inhibit the phosphorylation of MAPK-JNK and Akt (Thr308), and upregulate ESR1	Yu et al. (2023)
		luteolin and erastin	Induced iron apoptosis	HIC1 expression was upregulated and GPX4 expression was enhanced	Zheng et al. (2023)
	colon cancer	luteolin	Induced apoptosis	Antioxidant activity and MAPK signal transduction activation	Kang et al. (2017)
		luteolin	Inhibit migration and invasion	Upregulated miR-384 and downregulated PTN expression	Yao et al. (2019)
	colorectal cancer	luteolin	Decrease cell proliferation	regulate MicroRNA-301-3p	Moeng et al. (2020)
	pancreatic cancer	luteolin	Induced apoptosis	Targeting BCL-2 inhibits SW1990 cell death	Li et al. (2018)
		lopeol and stigmasterol	Inhibit tumor angiogenesis	Decrease TNF-α expression and affect VEGFR-2 signaling pathway	Kangsamaksin et al. (2017)
	Cholangiocarcinoma	stigmasterol	Induced apoptosis	Upregulated p27 expression and downregulated Jab1 expression	Pandey et al. (2019)
	Gall Bladder Carcinoma	lupeol and stigmasterol	Inhibit migration and invasion	Reduce TNF-α expression and affect VEGFR-2 signaling pathway	Kangsamaksin et al. (2017)
		luteolin	Inhibit migration; Induced apoptosis	Inhibition of SIRT1; ROS/JNK pathway mediated mitochondrial apoptosis pathway activation	Qin et al. (2021)
	esophageal carcinoma	stigmasterol	Induced apoptosis	Decreased Bcl-2 and BCL-XL gene expression	AmeliMojarad et al. (2022)
Reproductive and the urinary system cancer	breast cancer	Codonolactone	Inhibit cell invasion, migration and metastasis	Downregulated Runx2 transcriptional activity and inhibited MMPs	Wang et al. (2014)
		luteolin	Inhibit cell growth and EMT, induce apoptosis	Upregulated miR-203 expression and inhibited Ras/Raf/MEK/ERK signaling	Gao et al. (2019)
		luteolin	Inhibit cell migration and invasion	Down-regulating β-catenin and reversing EMT	Lin et al. (2017)
	triple-negative breast cancer	luteolin	Inhibit cell migration and invasion	Inhibiting YAP/TAZ activity	Cao et al. (2020)
		luteolin	Inhibit cell proliferation and metastasis	AKT/mTOR signaling pathway is inhibited to downregulate MMP9 expression	Wu et al. (2021)
		Stigmasterol	Inhibit cell proliferation, cycle and migration	Mitochondrial depolarization and ROS production, increase of calcium level and inhibition of MMPs expression	Bae et al. (2020)
	Ovarian Cancer	Codonopsis saponin A	Inhibit cell invasion and metastasis	Activate the ROS-mediated p38 pathway; inhibit MMP expression	Ahn et al. (2020)
		luteolin	Inhibit cell proliferation, induce apoptosis and G2/M cycle arrest	Activate the p53 signaling pathway	Chang et al. (2023)
		luteolin	Inhibition of cell invasion	Mediated AKT/mdm2 pathway upregulated E-cadherin expression	Zhou et al. (2009)
	prostate cancer	luteolin	Inhibit cell proliferation, migration and self-renewal	Upregulation of FZD6 inhibits Wnt signaling to inhibit PCa dryness	Han et al. (2018)
		luteolin	Inhibit cell proliferation and induce cell apoptosis	Androgen receptor (ARs) downregulation	Chiu and Lin (2008)
		luteolin	Inhibit cell proliferation	Upregulated TRX1, P21 and downregulated mTOR signal transduction	Iida et al. (2020)
	bladder cancer	Stigmasterol	Enhanced sensitivity to cisplatin	Inhibit Nrf2 signaling pathway	Liao et al. (2020)
	endometrial cancer	luteolin	Induced apoptosis	Enhanced histone H3 acetylation and c-Jun activation to stimulate Fas/FasL	Wang et al. (2018)
Other system cancer	leukemia	luteolin	Inhibit cell proliferation and induce apoptosis	Inhibition of RSK1 pathway	Deng et al. (2017)
		luteolin	Inhibit the proliferation and metastasis of osteosarcoma	The expression of AKT1, STAT3, IL6, TNF and VEGFA was decreased	Huang et al. (2023)
	osteosarcoma	luteolin	Inhibit cell proliferation and induce apoptosis	Promote the expression of BCL-2, Caspase-3 and Survivin proteins, and downregulate the expression of BAX protein level	Wang et al. (2015)
		luteolin	Inhibit cell migration	Inhibition of p-IGF-1R/PI3K/AKT/mTOR pathway activation	Wang et al. (2017a)
	glioblastoma			Downregulated Cdc42 expression and PI3K/AKT activity	Cheng et al. (2013)
		luteolin	Induced apoptosis	Activate ER stress and mitochondrial dysfunction	Wang et al. (2017b)

### 5.1 Application in respiratory system cancer

**FIGURE 5:**
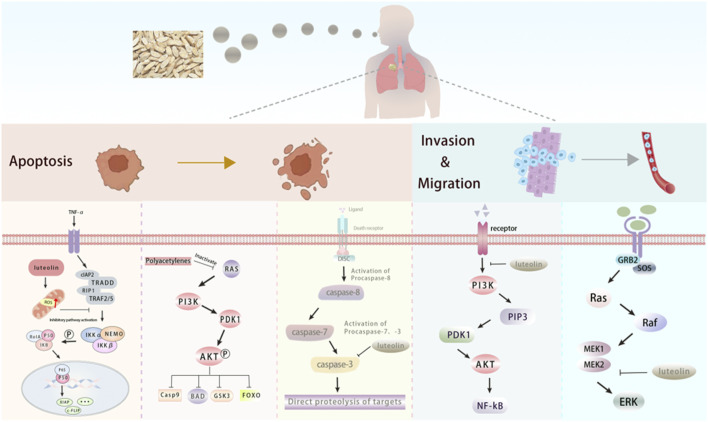
The active components of Radix Codonopsis promote lung cancer cell death and inhibitinvasion and metastasis. luteolin and Polyacetylenes can promote the apoptosis of lung cancercells by inhibiting PI3K/AKT, NF-kB and Caspase pathways. luteolin also attenuated EMT byinhibiting PI3K/AKT/NF-kB pathway and activating MEK/RK signaling pathway.

Lung cancer, a malignant tumor originating from lung cells, is the leading cause of cancer-related death worldwide. It is mainly categorized into small-cell lung cancer and non-small-cell lung cancer, with the incidence being significantly higher in men than in women (Sung et al., 2021). The active components of Radix Codonopsis can influence lung cancer by inhibiting tumor cell proliferation and promoting apoptosis, as illustrated in Figure 5. Additionally, Radix Codonopsis has therapeutic effect for nasopharyngeal carcinoma.

Polyacetylenes from Radix Codonopsis may induce apoptosis in lung adenocarcinoma cells by inhibiting the Ras/PI3K/AKT pathway, while also improving lung ecological imbalance by increasing microbial diversity and inhibiting common lung pathogens. At the same time, the study found that polyacetylenes did not affect the proliferation of human normal lung epithelial cells (Wang et al., 2022), indicating minimal side effects and strong clinical potential for lung cancer treatment. Luteolin, another bioactive compound in Radix Codonopsis, promotes apoptosis and inhibits migration of A549 lung adenocarcinoma cells by activating the MEK-ERK signaling pathway (Meng et al., 2016). Ma et al. (2015) studied the effect of luteolin on human large-cell lung cancer (NCI-H460) cells and found that luteolin could induce apoptosis of NCI-H460 cells, and its mechanism may be related to the downregulation of Sirt1 expression. The effect was further confirmed by lentivirus knockdown of Sirt1 protein level in NCI-H460 cells. Chen et al. (2013) observed the effect of luteolin on EMT of lung adenocarcinoma A549 cells. The results showed that luteolin inhibited the migration, invasion, and EMT ability of lung adenocarcinoma A549 cells. The mechanism may be related to the inhibition of the TGF-β1-induced EMT process in human lung cancer cells and further indicated that the PI3K/AKT/NF-kB/Snail/E-cadherin signaling pathway is closely related to TGF-β1-mediated EMT. It was found that luteolin significantly attenuated hypoxia-induced EMT in human non-small cell lung cancer cells. Further studies have found that luteolin inhibited the expression of integrin β1 and FAK, combined with the relationship between EMT and integrin β1 and FAK signal transduction, indicating that luteolin inhibited the EMT of human non-small cell lung cancer cells partly due to the inhibition of integrin β1 and FAK expression (Ruan et al., 2012). Yu et al. (2019) found that luteolin reduced the expression of AIM2 at both mRNA and protein levels in the study of the relationship between luteolin interfering with melanoma 2 (AIM2) and non-small cell lung cancer, thus inducing G2/M phase arrest and inhibiting EMT in non-small cell lung cancer (NSCLC). Further analysis revealed that luteolin’s anti-cancer effects in NSCLC are closely associated with AIM2 expression. Specifically, AIM2 knockdown diminished luteolin’s ability to exert its anti-tumor effects, while AIM2 overexpression reinforced luteolin’s therapeutic impact. These findings highlight the critical role of AIM2 in mediating luteolin’s efficacy against NSCLC. In addition, Lee et al. (2017) showed that luteolin can inhibit the proliferation of non-small cell lung cancer parental cells and drug-resistant cells, and the mechanism may be achieved by inhibiting the expression of TAM RTKs, rather than mediating the expression of IL-8. In Lewis lung cancer, luteolin induces cancer cell apoptosis by activating caspase 9 and 3-mediated mitochondrial apoptosis pathways. In addition, inhibition of ERK and Akt pathways is also one of the mechanisms of luteolin against Lewis lung cancer (Kim et al., 2007). Studies have shown that luteolin can be used as a radiosensitizer for non-small cell lung cancer. Luteolin combined with ionizing radiation (IR) can enhance the apoptosis of cancer cells both *in vivo* and *in vitro*. The mechanism is related to the activation of the p38/ROS/caspase pathway, which is of great help for the development of new treatment techniques for patients with non-small cell lung cance (Cho et al., 2015). In addition, studies have also revealed that luteolin can be used to treat nasopharyngeal carcinoma (Xiong et al., 2022). A study has also confirmed that luteolin can inhibit the cell cycle of human nasopharyngeal carcinoma (NPC) cells in a time and dose-dependent manner. The mechanism is related to the Akt/GSK-3β/Cyclin D1 pathway, which inhibits the proliferation of cancer cells by eliminating the effect of insulin on this pathway (Ong et al., 2010).

In summary, the active components of Radix Codonopsis, particularly luteolin, exhibit significant therapeutic effects against respiratory cancers. These compounds exert their pharmacological effects by inducing cancer cell apoptosis and inhibiting proliferation, migration, and invasion, highlighting the broad application prospects and clinical value of Radix Codonopsis.

### 5.2 Application in digestive system cancer

Globally, gastric cancer ranks as the fifth most common malignancy. In 2020, an estimated 1.1 million new cases were reported worldwide, resulting in approximately 800,000 deaths (Bray et al., 2024). These data highlight the seriousness of gastric cancer worldwide. The natural compounds extracted from Radix Codonopsis have a certain role in the treatment of gastric cancer. The active ingredient of Radix Codonopsis Stigmasterol belongs to sterol compounds and is a kind of plant sterol. Studies have shown that Stigmasterol can inhibit the proliferation of gastric cancer cells, and induce apoptosis and autophagy by blocking the AKT/mTOR signaling pathway, and autophagy has a protective effect on apoptosis. This study also showed that Stigmasterol could inhibit the growth of gastric cancer *in vivo*, suggesting that Stigmasterol may be a potential drug for the treatment of gastric cancer (Zhao et al., 2021). Taraxerol is another active ingredient of Radix Codonopsis, which is mainly found in plants, especially in Asteraceae plants such as dandelion. Huo et al. revealed the regulatory mechanism of taraxerol in gastric cancer through network pharmacology and verified the role of taraxerol in gastric cancer and its key signaling pathways through a series of *in vitro* experiments. Experiments have shown that taraxerol can inhibit the proliferation, migration, and invasion of gastric cancer cells, and induce cell cycle arrest and apoptosis. Its anti-gastric cancer effect is likely to be achieved by inhibiting the PI3K/AKT signaling pathway (Huo et al., 2022). However, this study was only verified at the cellular level, and further animal experiments are needed to prove its mechanism. Zang et al. (2017) showed that luteolin could reduce the migration and proliferation of human umbilical vein endothelial cells (HUVECs) in a dose-dependent manner, and significantly inhibit the tube formation and angiogenesis induced by gastric cancer cells. This inhibitory effect was achieved by reducing the secretion of VEGF and interfering with the interaction between gastric cancer cells and HUVECs, especially by down-regulating Notch1 expression. Overexpression of Notch1 partially reversed the effect of luteolin, confirming that luteolin inhibited gastric cancer angiogenesis and gastric cancer cell-derived tube formation through the Notch1-dependent pathway. Another study (Lu et al., 2015) showed that luteolin significantly inhibited tumor growth, reduced invasiveness, and promoted apoptosis in cMet-overexpressing gastric cancer models. The mechanism may be related to the inhibition of the cMet/Akt/ERK signaling pathway. Therefore, luteolin may be a potential drug for the treatment of cMet-overexpressing gastric cancer. Wu et al. (2015) found that luteolin affected the apoptosis of gastric cancer cells by regulating Bcl-2 protein. This experiment showed that the expression of miR-34a was downregulated in gastric cancer tissues, while luteolin could upregulate the expression of miR-34a and reduce the level of Bcl-2, which could be partially reversed by anti-miR-34a oligonucleotide. Therefore, the study revealed the important role of luteolin in inducing apoptosis of gastric cancer cells through the miR-34a pathway. In addition, lobetyolin extracted from Radix Codonopsis can inhibit the proliferation of gastric cancer cells and induce apoptosis. Its mechanism may be related to reducing glutamine uptake, down-regulating ASCT2 expression, inducing ROS accumulation, regulating AKT/GSK3β/c-Myc signaling pathway and reducing Nrf2 protein level, thus playing an anti-cancer role in gastric cancer (Cheng et al., 2023). In the study of Zang et al. (2019), it was shown that glycitein, an isoflavone compound contained in Radix Codonopsis, has a significant anti-tumor effect on human gastric cancer cells. It can play a role by increasing ROS production, reducing mitochondrial transmembrane potential, and inducing apoptosis and G0/G1 phase cell cycle arrest. The mechanism involves the activation of the MAPK signaling pathway and the inhibition of STAT3 and NF-κB signaling pathways, suggesting that glycitein may become a new targeted drug for the treatment of human gastric cancer. In summary, various active ingredients of Radix Codonopsis show significant potential and effects in anti-gastric cancer through different mechanisms of action.

**FIGURE 6:**
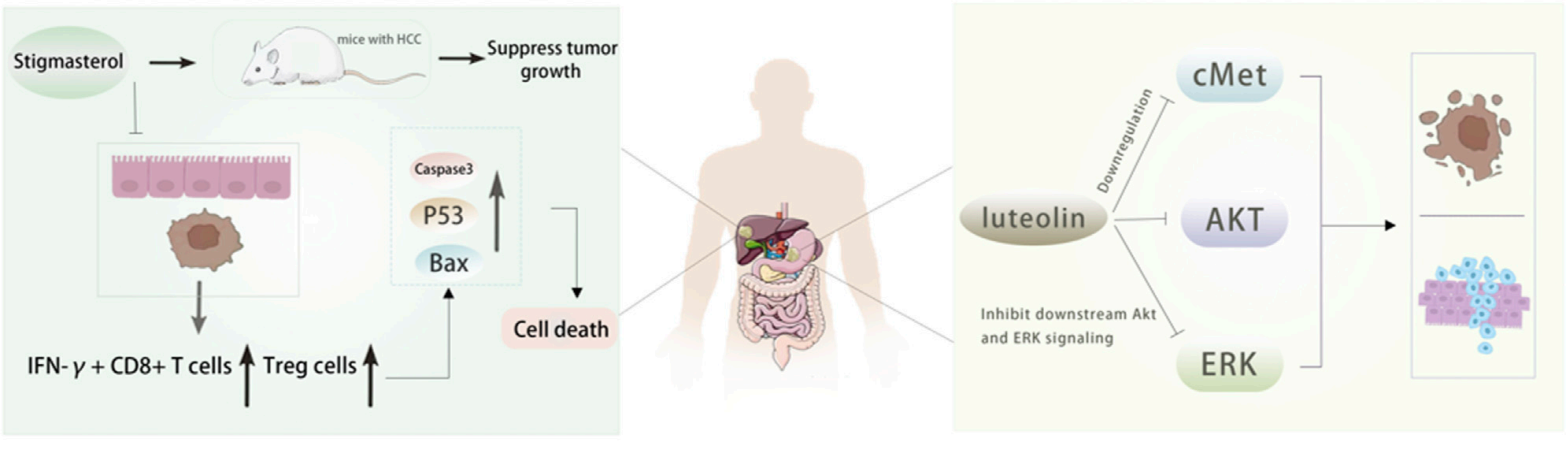
The active ingredients of Radix Codonopsis exert anticancer effects in gastric and livercancer. Stigmasterol was shown to inhibit tumor growth in mice. It was found that theincrease of IFN-y+ CD8^+^ T cells and Treg cells and the upregulation of Caspase3, Bax and P53 expression led to the death of tumor cells. luteolin can inhibit the proliferation, invasionand promote apoptosis of gastric cancer cells by inhibiting cMet/Akt/ERK signaling pathway.

Luteolin also has a good effect on anti-hepatocellular carcinoma. Through network pharmacology, 21 effective components and 98 potential target genes of Radix Codonopsis were screened, and 53 interacting genes were obtained by intersection with hepatocellular carcinoma (HCC) target genes. It was found that luteolin had an anti-hepatoma effect by affecting the ESR1 signaling pathway (Yu et al., 2023). In addition, Hwang et al.found that luteolin inhibited the growth of liver cancer cells and reduced tumor volume by activating AMPK, which may be related to the inhibition of NF-κB activity, indicating that AMPK is a potential target for cancer prevention (Hwang et al., 2011). A study (Huo et al., 2024) confirmed the inhibitory effect of Stigmasterol on tumor growth in HCC mice and found that it increased the proportion of IFN-γ + CD8 + T cells and Treg cells in intestinal mucosa and tumor tissues by affecting specific *Lactobacillus* species, resulting in increased apoptotic protein levels and tumor cell death, revealing the effect of Stigmasterol remodeling of intestinal flora on immune cells, which provides a theoretical basis for the clinical application of Stigmasterol in the treatment of hepatocellular carcinoma. Figure 6. In addition, the researchers determined the potential targets of Radix Codonopsis in hepatocellular carcinoma by screening the target proteins and key genes related to hepatocellular carcinoma. Through a series of experimental analyses, including cell experiments and animal models, it was found that Radix Codonopsis hair polysaccharide solution (CPP) could inhibit the proliferation, migration, and stem cell characteristics of liver cancer cells and induce apoptosis. The results show that Radix Codonopsis may inhibit the growth of hepatocellular carcinoma by regulating the CDK1/PDK1/β-catenin signaling axis, which provides a new insight into the role of Radix Codonopsis in the treatment of hepatocellular carcinoma (Li et al., 2024). Bai et al. studied two polysaccharides CPP1a and CPP1c isolated from Radix Codonopsis and confirmed that they were cytotoxic to HepG2 cells, inhibited cell migration, and induced apoptosis (Bai et al., 2018).

In the study of gallbladder cancer and cholangiocarcinoma, some components of Radix Codonopsis also have a certain therapeutic effect on them. The effect of Stigmasterol on cell proliferation was evaluated by MTT and trypan blue test, and its regulation on the expression of Jab1 and p27 in human gallbladder cancer cells was detected by RT-PCR and Western blotting. Further analysis showed that Stigmasterol could induce apoptosis and activate the mitochondrial apoptosis signaling pathway. The results showed that Stigmasterol has the potential as a therapeutic agent for gallbladder cancer targeting Jab1 (Pandey et al., 2019). Lupeol and stigmasterol have anti-inflammatory and potential anti-cancer effects. Studies have found that they can inhibit endothelial cell viability and migration, reduce TNF-α expression, affect the VEGFR-2 signaling pathway, and destroy tumor angiogenesis *in vivo* experiments. Reduce cholangiocarcinoma (CCA) tumor growth (Kangsamaksin et al., 2017).

Luteolin, as a natural biological flavonoid, shows anti-cancer and apoptosis-inducing properties in HCT-15 colon cancer cells *in vitro* experiments. It can reduce cell viability, affect cell cycle and apoptosis-related protein expression, and may become a potential drug for the prevention or treatment of colon cancer. Yao et al. (2019) have shown that luteolin has an anti-cancer effect in colorectal cancer and can inhibit cell migration and invasion. The mechanism may be related to the upregulation of miR-384 and downregulation of pleiotropic protein (PTN) expression, suggesting that PTN may become a potential target for CRC treatment. It has also been found that luteolin can selectively reduce the viability of HT-29 colon cancer cells and induce their apoptosis, which involves the activation of the mitochondrial-mediated caspase pathway and the activation of antioxidant enzymes. Luteolin induces apoptosis by regulating the expression of Bax and Bcl-2, promoting the release of cytochrome c, increasing the activity of caspase-9 and caspase-3, and increasing the level of reduced glutathione and the expression of GSH synthase, which is mediated by mitogen-activated protein kinase signaling pathway (Kang et al., 2017). In addition, the resistance of colon cancer to the ferroptosis inducer erastin is a therapeutic problem. The study found that the combination of luteolin and erastin showed a synergistic inhibitory effect on colon cancer cells *in vitro* and *in vivo*, and enhanced the sensitivity of colon cancer cells to ferroptosis by reducing the expression of antioxidant enzyme GPX4 and up-regulating the expression of tumor suppressor HIC1 (Zheng et al., 2023).

In addition to the above-mentioned cancers, the effective anti-cancer components of Radix Codonopsis can also play a role in other cancers of the digestive system. For example, in oral cancer, the study of Tiioe et al.aimed to find approved drugs with selective toxicity to oral squamous cell carcinoma (OSCC). Through drug screening, it was found that luteolin, metixene hydrochloride, and nitazoxanide had high cytotoxicity to oral cancer cells, and the toxicity was lower than the existing standard drugs. Luteolin can affect the DNA repair pathway and may be used as a potent cytotoxic or adjuvant therapy for oral cancer. However, further *in vivo* studies are needed to verify the anti-cancer effect of luteolin (Tjioe et al., 2016). Paclitaxel as an anti-tumor drug has application limitations, but it may be effective in combination with other drugs. It was found that luteolin combined with low-dose paclitaxel showed synergistic anti-esophageal cancer effects *in vitro* and *in vivo*, and played a role by inhibiting SIRT1 and ROS/JNK pathways without obvious toxicity (Qin et al., 2021). Luteolin, as a natural small molecule inhibitor of BCL-2, shows pro-apoptotic activity in SW1990 pancreatic cancer cells promotes BAX release, and induces cancer cell death by directly binding to BCL-2. *In vivo* experiments, luteolin can also effectively inhibit tumor growth (Li et al., 2018). Moeng et al.’s study revealed that miR-301-3p plays a key role in regulating cell proliferation and enhancing the antitumor effect of tumor necrosis factor-related apoptosis-inducing ligand (TRAIL), and TRAIL can inhibit cell growth by inducing cell cycle arrest. Luteolin can inhibit the growth of human pancreatic cancer PANC-1 cells and increase their sensitivity to TRAIL by reducing the level of miR-301-3p (Moeng et al., 2020).

In summary, a variety of active ingredients in Radix Codonopsis, such as luteolin and Stigmasterol, can exert their corresponding anti-tumor effects on various cancers of the digestive system. Its application is expected to bring more benefits to patients with digestive system cancer.

### 5.3 Application in reproductive system and urinary system cancer

In recent years, the effective components of Radix Codonopsis have been studied, and it has been found that it also has potential curative effects on reproductive and urinary system cancers, such as prostate cancer and cervical cancer, which provides a direction for the in-depth study of Radix Codonopsis in the field of anti-cancer.

Breast cancer is a cancer that occurs in breast tissue, mainly affecting women, but it can also occur in men. Its incidence is rising worldwide. Therefore, it is necessary to carry out continuous research on it and constantly develop new therapeutic drugs and methods. A study evaluated the effect of Stigmastero on the expression of anti-apoptotic genes Bcl-2 and BCL-XL in human breast cancer MCF-7 cell line, as well as its effect on apoptosis, cell viability, and spontaneous breast tumor growth *in vivo*. The results showed that Stigmastero could significantly reduce the expression of these genes, induce apoptosis, reduce cell proliferation, and inhibit tumor growth (AmeliMojarad et al., 2022). Gao et al.showed that luteolin inhibited the growth and EMT of breast cancer cells. It exerts anti-tumor effects by reducing cell viability, accelerating apoptosis, regulating related protein expression, and inhibiting TGFβ1-induced EMT process. In addition, the anti-tumor effect of luteolin is related to the increase of miR-203 level and the inhibition of Ras/Raf/MEK/ERK signaling pathway, and miR-203 silencing will weaken its effect (Gao et al., 2019). Codonolactone (CLT) is a sesquiterpene lactone isolated from Radix Codonopsis. Wang et al. (2014) found that CLT can reduce the invasion and migration of breast cancer cells, and significantly inhibit the formation of lung metastases of breast cancer *in vivo*. Further studies have shown that this anti-metastasis effect may be related to CLT inhibition of MMP-9 and MMP-13 activity and expression, as well as downregulation of Runx2 transcriptional activity. Triple-negative breast cancer (TNBC) is associated with a high risk of early recurrence and metastasis, and EMT plays a key role in its development. Abnormal activation of YAP/TAZ promotes EMT. A study has shown that luteolin can inhibit YAP/TAZ activity and reverse EMT, and reduce TNBC cell migration and tumor growth, suggesting that luteolin may become a new drug for the treatment of TNBC (Cao et al., 2020). In addition, luteolin was found to have an anti-metastasis effect in TNBC, which could inhibit the migration and invasion of highly metastatic TNBC cells in a dose-dependent manner and reverse EMT. *In vivo* experiments, luteolin significantly reduced lung metastasis of breast cancer and inhibited the expression of EMT-related molecules and β-catenin. The results showed that luteolin effectively inhibited breast cancer metastasis by down-regulating β-catenin-mediated EMT reversal (Lin et al., 2017). Another study evaluated the therapeutic effect and mechanism of luteolin on androgen receptor-positive breast cancer cells. It was found that luteolin could significantly inhibit the proliferation and metastasis of these cells, and this effect was achieved by affecting the AKT/mTOR signaling pathway and reducing the expression of MMP9. The use of luteolin combined with AKT/mTOR inhibitors can enhance its inhibitory effect (Wu et al., 2021).

Ovarian cancer is one of the most lethal types of cancer in the female reproductive system. It often has no obvious symptoms in the early stage, so regular physical examination and improvement of prognosis are crucial. As a phytosterol, Stigmastero also has an anti-cancer effect on human ovarian cancer. Bae et al. (2020) found that Stigmastero can inhibit the growth of ovarian cancer cells, induce apoptosis, affect mitochondrial function and calcium levels, and inhibit cell migration and angiogenesis. Radix Codonopsis saponin A, the main triterpenoid saponin in the roots of Radix Codonopsis, can significantly inhibit the migration and invasion of human ovarian cancer cells and reduce the expression of matrix metalloproteinases (MMPs) -2 and -9. The mechanism may be related to the production of ROS and the activation of p38 MAP kinase (Ahn et al., 2020). There are also studies aimed at exploring the effect of luteolin as a new therapeutic drug targeting vaccine-associated kinase 1 (VRK1) on high-grade serous ovarian cancer (HGSOC). It was found that luteolin could reduce the proliferation of HGSOC cells, increase apoptosis and G2/M phase cell cycle arrest, and activate the p53 signaling pathway. In patient-derived xenograft models, oral or intraperitoneal injection of luteolin significantly inhibited tumor growth and showed a synergistic effect with cisplatin in combination therapy, especially for cisplatin-resistant cell lines. Therefore, luteolin is considered a promising candidate for HGSOC treatment (Chang et al., 2023). In addition, endometrial cancer is also one of the most common cancers in women. It is a malignant tumor originating from endometrial epithelial cells. However, the chemotherapy resistance of patients with endometrial cancer reduces their survival rate, and Nrf2 plays an important role in chemotherapy resistance. Chang et al.found that Nrf2 was highly expressed in endometrial cancer tissues, resulting in decreased sensitivity to cisplatin. As a new Nrf2 inhibitor, Stigmastero can improve the sensitivity of cancer cells to cisplatin and reduce the level of Nrf2 protein, thus playing a key role in overcoming chemotherapy resistance (Liao et al., 2020).

Prostate cancer is a very common malignant tumor in elderly men. In recent years, with the aging of China’s population and lifestyle changes, the incidence of prostate cancer in China is also gradually increasing. Luteolin was found to inhibit the invasion of prostate cancer cells by regulating the expression of E-cadherin. Studies have shown that luteolin affects E-cadherin through the AKT/MDM2 pathway, and can inhibit the lung metastasis of prostate cancer cells in nude mice *in vivo* (Zhou et al., 2009). In addition, Han et al. (2018) found that luteolin can reduce the proliferation, migration, self-renewal, and stem cell marker expression of prostate cancer cells *in vitro*. FZD6 is a tumor suppressor that can eliminate prostate cancer stemness. Further studies have shown that luteolin inhibits Wnt signaling by up-regulating FZD6 to inhibit prostate cancer stemness. Chiu and Lin’s study (Chiu and Lin, 2008) also revealed that luteolin can inhibit the proliferation of prostate cancer cells and induce apoptosis of prostate cancer LNCaP cells, while reducing prostate-specific antigen (PSA) level and Androgen receptor (AR) expression, leading to AR degradation by reducing the association between AR and heat shock protein 90, indicating that luteolin plays an anti-cancer role by targeting AR and may be used for the chemoprevention and treatment of prostate cancer.

In the study of Iida et al. (2020), it was found that luteolin may become a natural product therapeutic agent for the treatment of bladder cancer. Luteolin can inhibit the survival of human bladder cancer cell line T24 by regulating p21, TRX1, and mTOR signaling pathways, inducing cell cycle arrest, and reducing reactive oxygen species production. In animal models, luteolin significantly reduced the volume of bladder tumors and decreased the expression of phosphor (p) -S6 downstream of Ki67 and mTOR. This study also showed that luteolin metabolites were associated with inhibition of cell proliferation and mTOR signal transduction. Therefore, the effective components of Radix Codonopsis also have a good application in the treatment of reproductive and urinary system cancer.

### 5.4 Application in other system cancer

Modern pharmacological studies have shown that Radix Codonopsis not only has a positive effect on respiratory, digestive, reproductive, and urinary system cancers but also shows potential therapeutic effects on other types of tumors, such as leukemia, osteosarcoma, and other malignant tumor diseases.

Leukemia, also known as blood cancer, is a type of malignant tumor that affects blood and bone marrow. Improving its therapeutic effect and providing better treatment for patients is the goal pursued by researchers and medical workers. Wang et al.found that luteolin could significantly reduce the viability of human leukemia cells by inducing poly (ADP-ribose) polymerase (PARP) cleavage, nuclear fragmentation, and activating exogenous apoptotic pathways. This process is associated with upregulation of Fas/FasL expression, increased histone H3 acetylation, and activation of the c-Jun signaling pathway (Wang et al., 2018). In addition, the study found that RSK1 is overexpressed in bone marrow samples of untreated leukemia patients and is associated with shorter overall survival. Luteolin, as a novel RSK inhibitor, has been shown to inhibit growth, induce apoptosis, and reduce migration in leukemia cells. These effects of luteolin are related to the expression level of RSK1, and knockdown of RSK1 expression can enhance the effect of luteolin (Deng et al., 2017).

The incidence of osteosarcoma is high in adolescents and young adults, which has a significant impact on the physical and mental health and quality of life of patients. Therefore, it is necessary to study it to improve the therapeutic effect and the survival rate of patients. A study explored how luteolin induces apoptosis of MG-63 human osteosarcoma cells by inhibiting the expression of BCL-2, Caspase-3, and Survivin and promoting the expression of BAX, thereby inhibiting cell proliferation. The results showed that luteolin effectively inhibited the proliferation of osteosarcoma cells and induced apoptosis in a dose-dependent manner, suggesting that it may be used as a new drug for the treatment of osteosarcoma (Wang et al., 2015). Huang et al.found that luteolin can target multiple targets of osteosarcoma through network pharmacology analysis, especially key genes in the PI3K-AKT signaling pathway, such as AKT1, IL6, JUN, STAT3, TNF, and VEGFA. Experiments have confirmed that luteolin can inhibit the expression of these genes, reduce the viability of osteosarcoma cells, and inhibit tumor proliferation and metastasis in mouse models, indicating that luteolin may inhibit osteosarcoma by regulating related signaling pathways (Huang et al., 2023).

The study also found that luteolin can significantly inhibit the migration of human glioblastoma cells, which is related to the downregulation of MMP-2 and MMP-9 and the upregulation of TIMP-1 and TIMP-2, and affects epithelial-mesenchymal transition and p-IGF-1R/PI3K/AKT/mTOR signaling pathway. These results suggest that luteolin may be used for chemoprevention of glioblastoma (Wang et al., 2017a). In addition, studies have found that luteolin can inhibit the migration and invasion of glioblastoma cells at a non-toxic dose, and affect the level of Cdc42 protein and PI3K/AKT signaling pathway. These effects may be related to the promotion of Cdc42 protein degradation through the proteasome pathway (Cheng et al., 2013). Wang et al. (2017b) found that luteolin can induce apoptosis by increasing the level of intracellular reactive oxygen species, inducing endoplasmic reticulum stress response and mitochondrial dysfunction in human glioblastoma cell lines. The use of antioxidants can reverse this process. It indicates that luteolin may be an anticancer candidate for the treatment of glioblastoma.

## 6 Discussion and prospects

Cancer arises from dysregulated cell growth caused by genetic mutations. Its hallmark features—uncontrolled proliferation, evasion of apoptosis, induction of angiogenesis, and the capacity for invasion and metastasis—pose significant challenges for effective treatment. Consequently, developing safer and more effective anti-cancer therapies is of paramount importance. Recent studies have highlighted the potential of TCM and its bioactive components as anti-cancer agents, given their ability to exert synergistic, multi-component, multi-target, and multi-pathway regulatory effects. These components play roles in various tumor-related processes, including occurrence, progression, metastasis, and immune regulation. Radix Codonopsis, a TCM commonly recognized for both medicinal and nutritional value, has attracted attention for its broad range of pharmacological activities. In addition to its known benefits for cardiovascular and gynecological conditions, immune modulation, antioxidant and anti-aging effects, and fatigue resistance, Radix Codonopsis has shown promise in cancer treatment. However, no comprehensive review previously addressed its anticancer properties. Therefore, this paper reviews the research progress of the active ingredients of Radix Codonopsis in anti-tumor aspects, and summarizes in Table 2 that the active ingredients of Radix Codonopsis can play a role in various systems of cancer through multiple mechanisms of action. For example, some compounds can inhibit the proliferation, growth, and migration of cancer cells through the PI 3K/Akt signaling pathway, MEK/ERK signaling pathway, and p53 signaling pathway, and exert the therapeutic effect of Radix Codonopsis on various systems of cancer through multi-target and multi pathway approaches, in order to provide reference for future research and clinical applications.

Despite these promising findings, the mechanisms underlying the anticancer effects of Radix Codonopsis remain complex and warrant further investigation. Currently, the application of TCM in oncology often involves combining herbal therapies with conventional treatments to mitigate side effects and enhance therapeutic outcomes. While studies on luteolin and stigmasterol have advanced our understanding of Radix Codonopsis’s anticancer potential, other constituents—such as perlolyrine, frutinone A, and spinoside A—have been less explored. Further in-depth research is needed to fully elucidate their medicinal value, establish efficacy and safety profiles, and optimize their therapeutic use in cancer treatment.
